# Supplementary material for: A Humanized Bone Niche Model Reveals Bone Tissue Preservation Upon Targeting Mitochondrial Complex I in Pseudo-Orthotopic Osteosarcoma
Source: J Clin Med. 2019 Dec 11;8(12):2184. doi: 10.3390/jcm8122184 (PMC6947153; doi:10.3390/jcm8122184)
Supplement: Supplementary file 1 [file jcm-08-02184-s001.pdf]

Supplementary Figures

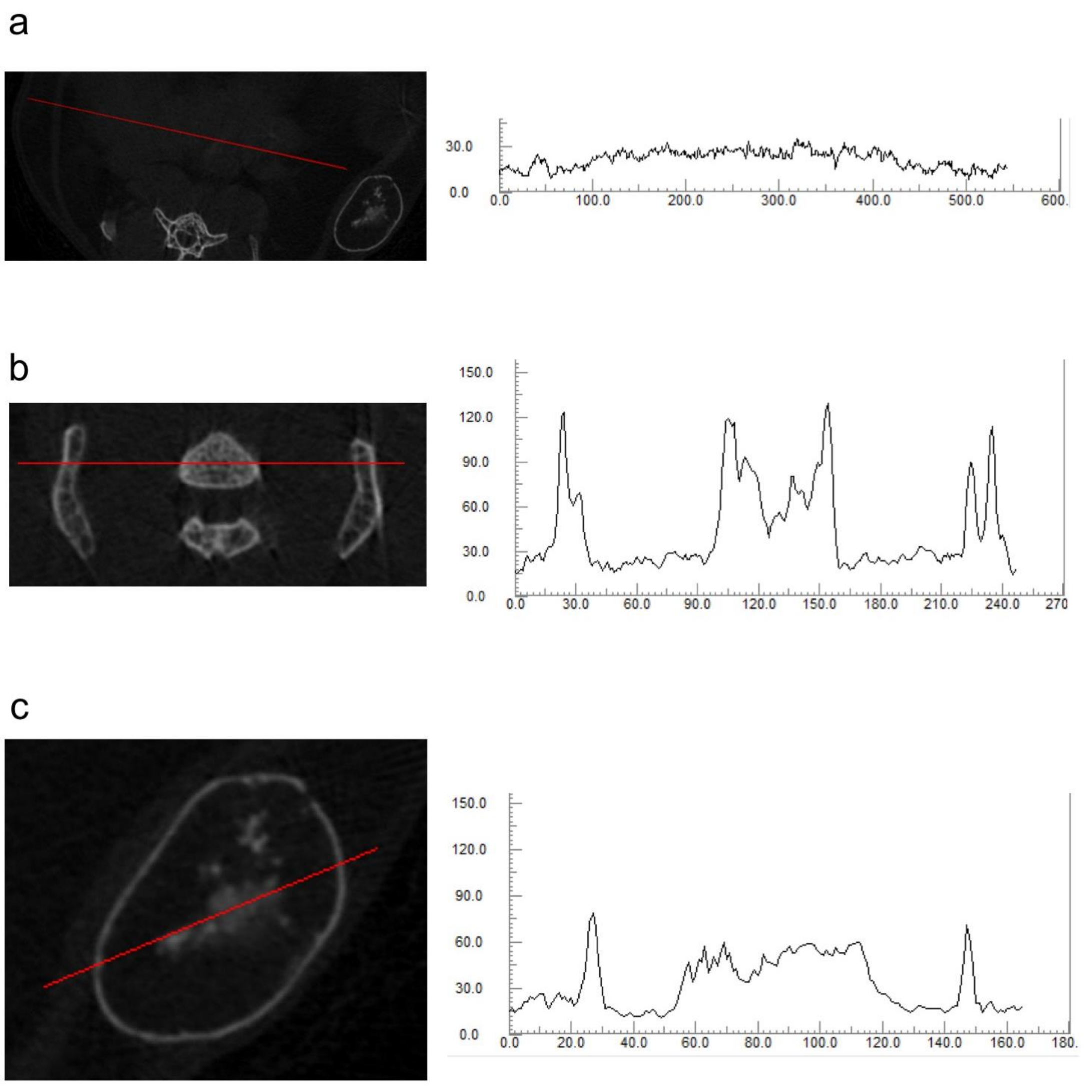

Supplementary Figure 1. Micro CT scans of the mouse body sections selecting (**a**) soft tissue, (**b**) bone tissue and (**c**) humanized bone niche control sample.

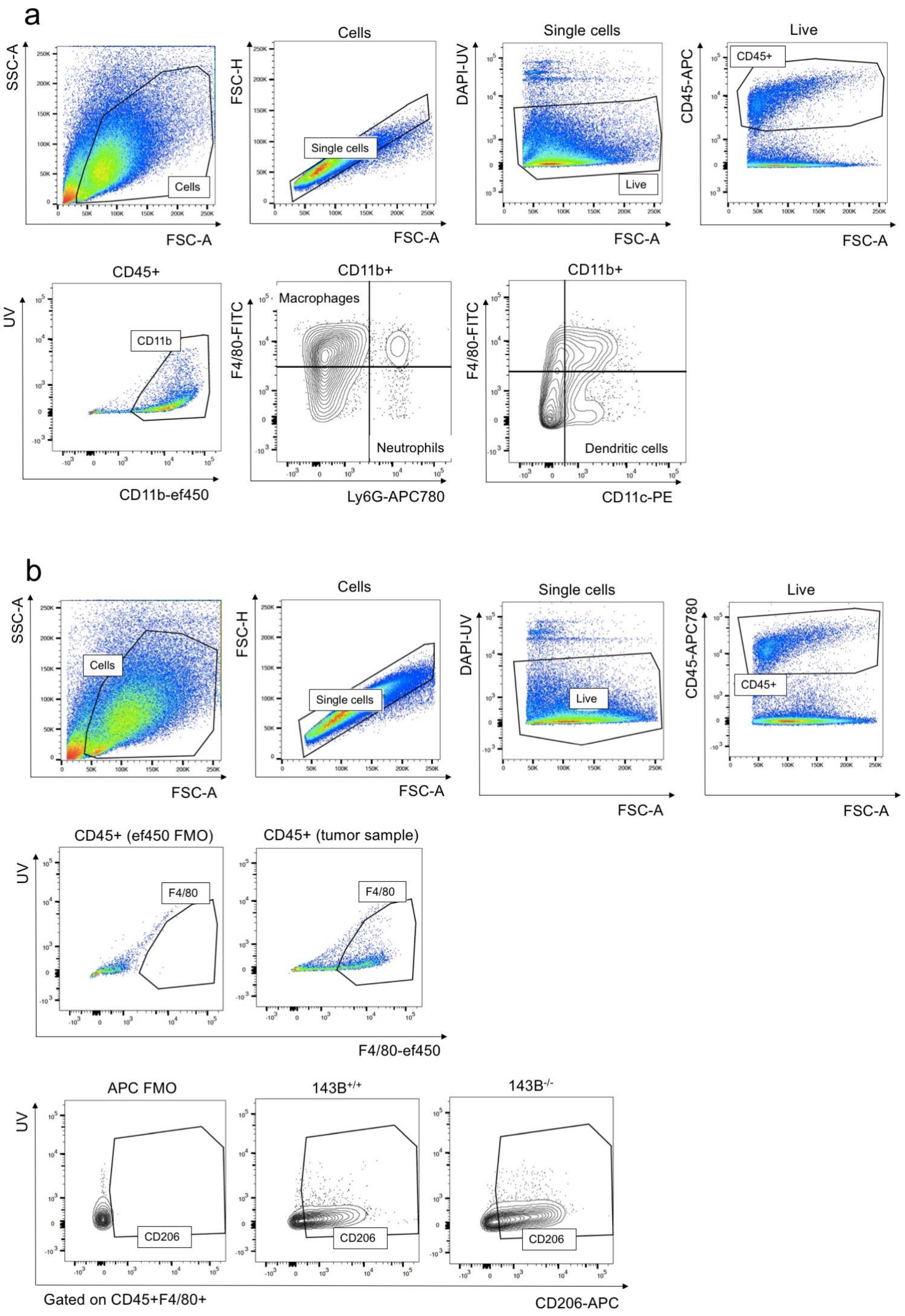

Supplementary Figure 2. Gating strategies for flow cytometry analyses of (**a**) innate immune system cell populations and (**b**) CD206+ macrophages.
